# Radiation pneumonitis following Yttrium-90 radioembolization: A Korean multicenter study

**DOI:** 10.3389/fonc.2023.977160

**Published:** 2023-01-16

**Authors:** Hyo-Cheol Kim, Gyoung Min Kim

**Affiliations:** ^1^ Department of Radiology, Seoul National University Hospital, Seoul National University College of Medicine, Seoul, Republic of Korea; ^2^ Department of Radiology, Yonsei University College of Medicine, Seoul, Republic of Korea

**Keywords:** hepatocellular carcinoma, radioembolization, radiation pneumonitis, dosimetry, dyspnea

## Abstract

**Objective:**

To report the incidence of radiation pneumonitis after radioembolization.

**Methods:**

In this retrospective study, from May 2009 to July 2021, 782 consecutive patients underwent radioembolization in two institutes. Medical internal radiation dose dosimetry and partition dosimetry were used for glass and resin Yttrium-90-labeled microspheres (^90^Y-microspheres), respectively. Medical records and radiological findings were retrospectively evaluated with emphasis on the symptomatic radiation pneumonitis.

**Results:**

Of the 732 patients with lung shunt study and follow-up, 13 (1.8%) had symptomatic radiation pneumonitis and six patients died due to radiation pneumonitis. Of the 721 patients whose lung doses were calculated, 10 patients who were treated with glass (n = 5) and resin (n = 5) ^90^Y-microspheres had radiation pneumonitis. No significant statistical difference between glass and resin ^90^Y-microspheres (p = 0.304) was noted in terms of radiation pneumonitis incidence. Among the patients with radiation pneumonitis, all five patients treated with glass ^90^Y-microspheres had estimated lung doses > 29 Gy, whereas five patients treated with resin ^90^Y-microspheres had relatively wide range of lung dose reaching much lower value (13.21Gy).

**Conclusion:**

The present study suggests that radiation pneumonitis after radioembolization may occur even though the manufacturer’s instructions are followed.

## 1 Introduction

Radioembolization, using ^90^Y-microspheres, are used for the treatment of malignant liver tumors (e.g., hepatocellular carcinomas (HCCs) and colorectal liver metastases) (1). Radioactive ^90^Y-microspheres are small enough to pass through tumoral vessels in rare patients, resulting in radiation pneumonitis. For this reason, planning angiography and simulating ^90^Y-microsphere delivery by infusing ^99m^Tc-macroaggregated albumin (^99m^Tc-MAA) into the hepatic artery are used to measure the lung shunt fraction (LSF) and the estimated lung dose (2). The 25-Gy estimated lung dose by partition dosimetry is believed to be the safe upper limit for resin ^90^Y-microspheres (3, 4). However, for glass ^90^Y-microspheres, radioembolization is relatively contraindicated when the estimated lung dose is >30 Gy in a session and 50 Gy in a lifetime by medical internal radiation dose (MIRD) dosimetry (5).

Radiation pneumonitis is a rare but serious radioembolization complication that can occur 1–6 months after the procedure. Several case reports of radiation pneumonitis have been noted in the literature (6–8). This study, herein, reports the incidence ofradiation pneumonitis after radioembolization in the Asian population.

## 2 Materials and methods

### 2.1 Patients

From May 2009 to July 2021, 782 consecutive patients underwent radioembolization in two institutes. The inclusion criteria were (a) patients who underwent planning angiography and ^99m^Tc-MAA scan and (b) patients whose information regarding lung dose was available. Exclusion criteria were (a) no lung shunt study (n = 37) and (b) no follow-up imaging for at least 2 months (n = 13). Consequently, 732 patients (mean age, 62.8 ± 12.4 years [range, 21–92 years]), which comprised 592 men and 140 women, were included in the present study. Among these patients, 664 and 68 had hepatocellular carcinoma and other cancers, respectively (**Table 1**). Moreover, 493 and 239 patients were treated with glass and resin ^90^Y-microspheres, respectively. Furthermore, 36 patients received two sessions and 10 patients received three sessions of radioembolization.

**Table 1:** Baseline characteristics in 732 patients who received radioembolization.

		Total (n = 732)	Radiation pneumonitis (n = 13)
Sex	Men	592	11
	Women	140	2
^90^Y-Microspheres	glass	493	6
	resin	239	7
Tumor type	HCC	664	13
	Colorectal cancer	31	
	Cholangiocarcinoma	23	
	Neuroendocrine tumor	7	
	Breast cancer	2	
	Gallbladder cancer	1	
	Gastrointestinal stromal tumor	1	
	Ampular of vater cancer	1	
	Gastric cancer	1	
	Hepatoblastoma	1	

### 2.2 Planning angiography and 99mTc-macroaggregated albumin imaging

Planning angiography includes celiac and superior mesenteric angiography, and depending on the need, right and/or left hepatic angiograms are also included. The operator advanced a microcatheter into the lobar artery supplying the primary target tumor, and 185 MBq of ^99m^Tc-MAA was injected into the lobar hepatic artery. After injecting ^99m^Tc-MAA, planar body scans that conjugated the anterior and posterior images were obtained for 10 min and were used to calculate the LSF.

### 2.3 Volume measurement and lung dose calculation

Total liver volume, target volume, and tumor volume of each patient were measured from the most recent cross-sectional imaging study before treatment, including computed tomography (CT) scan and magnetic resonance imaging using Aquarius Intuition (Terarecon, Durham, NC).

For Therasphere, single compartment dosimetry (MIRD) was used to calculate the estimated lung dose as provided by the manufacturer, and the lung mass was set as 1,000g for all patients. For SIR-Spheres, partition dosimetry provided by the manufacturer was used, and the lung weight was set as 800 and 600 g for men and women, respectively.

### 2.4 Analysis

Medical records and radiological findings were retrospectively evaluated. Radiation pneumonitis was diagnosed when patients presented with restrictive ventilatory dysfunction and typical bilateral lung infiltrates with exertional dyspnea and dry cough (9), and pathogens causing similar presentation such as pneumocystis carinii were not revealed. Severity was graded by Common Terminology Criteria for Adverse Events (CTCAE) v5.0. The chi-square test was used to compare the radiation pneumonitis incidences between the groups. A p value <0.05 was considered statistically significant. All statistical analyses were performed with SPSS version 25.0 software (SPSS, Inc., Chicago, IL, USA).

## 3 Results

**Figure 1:**
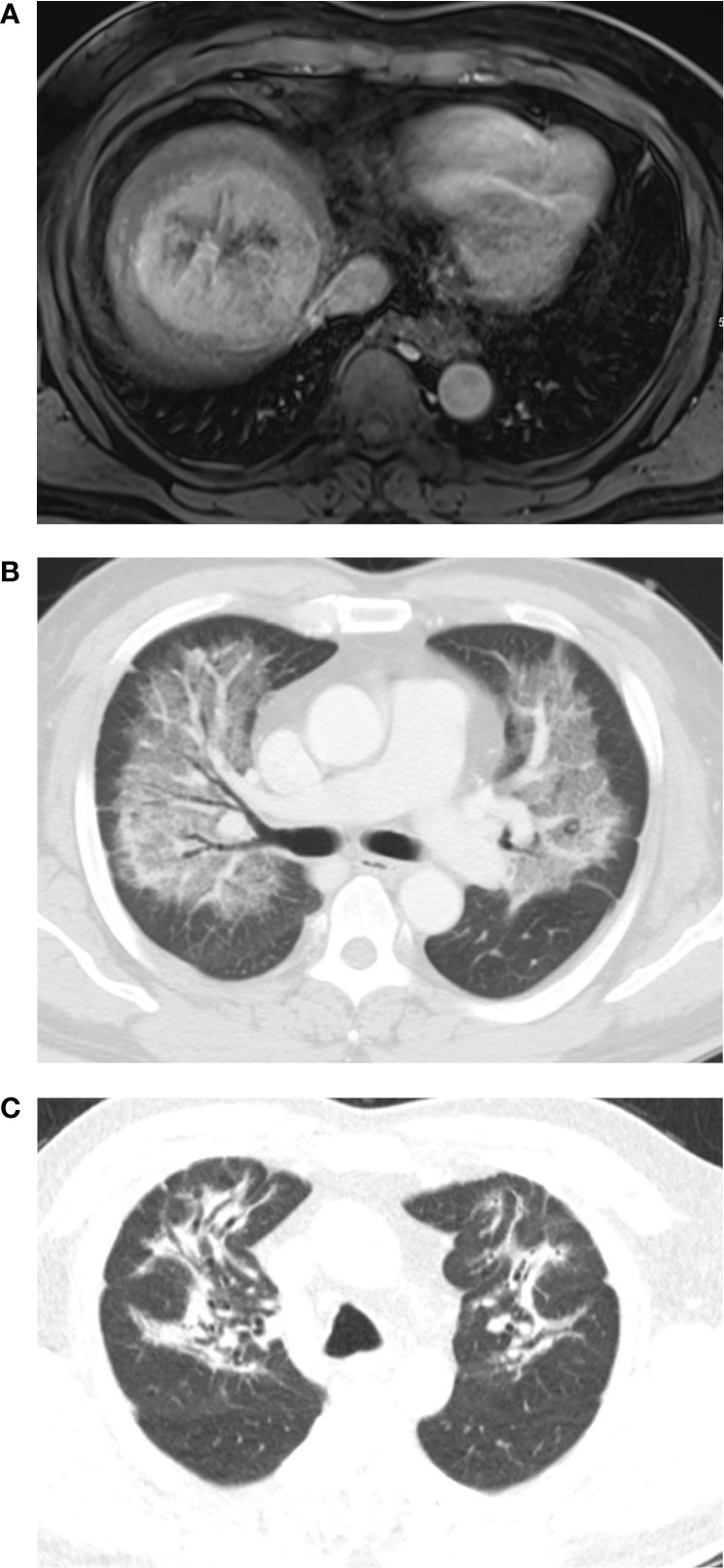
A 65-year-old man had a 9cm single mass in the right lobe. The lung shunt fraction was 14.1%, and 2.4 GBq of resin ^90^Y-microspheres were delivered into the right hepatic artery. The estimated lung dose was 21.01 Gy. **(A)** Magnetic resonance shows a mass in right lobe. **(B)** Chest CT scan 3.8 months after radioembolization shows diffuse consolidation and ground-glass opacity in both the lungs with subpleural sparing. **(C)** Chest CT scan 4 months after steroid therapy shows fibrotic changes in both the lungs.

Of the 732 patients, 13 (1.8%) had symptomatic radiation pneumonitis and were treated with steroids (**Table 2**) (**Figure 1**). All 13 patients did not receive systemic chemotherapy. All 13 patients had HCCs which were treated with glass (n = 6) and resin (n = 7) ^90^Y-microspheres. Dyspnea (n = 12) and cough (n = 6) were the common symptoms. The time interval between radioembolization and symptom onset ranged from 1.1 to 6.7 months (mean, 3.5 months; median, 3.3 months). Six patients died due to respiratory failure without tumor progression. Only one patient (patient no. 12) had radiation pneumonitis after second session of radioembolization, among the 46 patients who received multiple sessions.

**Table 2:** Treatment factors, symptom and outcome of radiation pneumonitis.

Sex/Age	^90^Y-microspheres used	Total radiation activity delivered (GBq)	Lung shunt fraction (%)	Estimated lung dose (Gy)	Symptom	Symptom onset after radioembolization (months)	Outcome	CTCAE grade	Follow-up period after diagnosis of radiation pneumonitis (months)
M/61	Glass	12.99	4.57	29.12	Dyspnea, cough	3.3	Improved	3	8
M/78	Glass	3.14	19.4	29.78	Dyspnea	1.3	Died	5	1
F/62	Glass	7.23	8.93	31.61	Dyspnea, cough	6.1	Died	5	2.5
M/84	Glass	7.37	10.0	36.15	Cough	6.0	improved	3	18
M/65	Glass	5.89	13.21	38.14	Dyspnea	5.5	Improved	3	12
F/71	Resin	2.7	5.91	13.21	Dyspnea, cough	1.6	Died	5	0.7
M/55	Resin	2.5	12.15	18.86	Dyspnea	4.3	improved	3	16
M/65	Resin	2.4	14.1	21.01	Dyspnea	3.8	improved	3	27
M/47	Resin	3.0	16.23	30.23	Dyspnea	1.1	Died	5	0.6
M/78	Resin	3.0	18.22	33.94	Dyspnea	6.7	improved	3	26
M/60	Glass	3.45	7.65	12.95*	Dyspnea, cough	2.8	Improved	3	9
M/91	Resin	1.8/1.5	14.38/not measured	16.07/13.39^#^	Dyspnea	3.6	Died	5	0.4
M/76	Resin	2.0	49.32	61.24^$^	Dyspnea, cough	3.3	Died	5	0.7

* The tumor was supplied by both the right hepatic artery and right inferior phrenic artery. ^99m^Tc-MAA was injected into only the right hepatic artery. Right inferior phrenic angiogram showed tumor blush and pulmonary shunt. ^90^Y-microspheres were injected into both right hepatic and right inferior phrenic arteries, assuming that the lung shunt fraction from the right inferior phrenic artery was same as that of the right hepatic artery.

# 2^nd^ session of radioembolization was performed 6 months after 1^st^ session of radioembolization without lung shunt study. It was assumed that lung shunt fraction at 2^nd^ session was 14.38%.

^$^ Hepatic angiogram shows a large arteriovenous shunt. ^99m^Tc-MAA was injected without embolization of arteriovenous shunt. ^90^Y-microspheres were injected after embolization of arteriovenous shunt.

Lung dose was not accurately calculated in 11 patients (three and eight with and without radiation pneumonitis, respectively). Radioembolization was performed by placing a balloon catheter in the right hepatic vein to reduce lung shunt in eight of 719 patients without radiation pneumonitis. Lung dose was not precisely estimated in the following three patients with radiation pneumonitis. Patient no. 11 had HCC, which was supplied by both the hepatic and right inferior phrenic artery. ^99m^Tc-MAA was injected into the right hepatic artery, and the LSF was 7.65%. The right inferior phrenic angiogram showed tumor blush and pulmonary shunt, and ^90^Y-microspheres were injected into both the right hepatic and right inferior phrenic arteries without arteriovenous shunt embolization. Patient no. 12 had received two radioembolization sessions. The LSF was 14.38% at the first radioembolization session. The second radioembolization session was performed 6 months after the first session, and the LSF was assumed as 14.38% without repeating the lung shunt study. Patient no.13 had a large arteriovenous shunt, and the LSF was 49.32%. ^90^Y-microspheres were injected into the hepatic artery after complete arteriovenous shunt embolization.

The lung dose could be calculated in 721 patients, which ranged from 0.013 to 38.14 Gy (**Table 3**). The estimated lung dose was >30 and >25 Gy in 24 and 20 patients who were treated with glass and resin ^90^Y-microspheres, respectively. Of the 721 patients, 10 who were treated with glass (n = 5) and resin (n = 5) ^90^Y-microspheres had radiation pneumonitis. No significant statistical difference between glass and resin ^90^Y-microspheres (p = 0.304) was noted in terms of radiation pneumonitis incidence. For glass ^90^Y-microspheres, radiation pneumonitis was more frequent in patients whose lung dose was >30 Gy than in patients whose lung dose was ≤30 Gy (p = 0.001). The same trend was obtained for resin ^90^Y-microsphere, though the difference was not statistically significant (p = 0.061) probably for an insufficient number of patients. Notably, all five radiation pneumonitis patients treated with glass ^90^Y-microspheres had estimated lung dose >29 Gy (29.12 ~ 38.14Gy). In contrast, five radiation pneumonitis patients treated with resin ^90^Y-microspheres had relatively wide range of lung dose reaching much lower level (13.21Gy).

**Table 3:** Baseline characteristics in 721 patients whose lung dose was able to be calculated.

			Total (n = 721)	Radiation pneumonitis (n = 10)	P value
Sex		Men	581	8	1.0
		Women	140	2	
^90^Y-Microspheres		glass	490	5	0.304
		resin	231	5
Lung dose	Glass ^90^Y-microspheres	≤ 30 Gy	466	2	0.001
		> 30 Gy	24	3
	Resin ^90^Y-microspheres	≤ 25 Gy	211	3	0.061
		> 25 Gy	20	2

## 4 Discussion

Radiation pneumonitis is rare but could be a fatal complication after radioembolization (6–8). Exertional dyspnea and dry cough are common clinical manifestations. CT scan commonly shows bilateral symmetric ground-glass opacity and consolidation with relative peripheral/hilar sparing, which is the so-called “bat wing appearance.” In addition, steroid therapy is the treatment mainstay.

MIRD dosimetry for glass ^90^Y-microspheres is used, and 30 Gy of lung dose is considered as an upper limit. Salem et al. reported no case of radiation pneumonitis in 403 patients treated with glass ^90^Y-microspheres (10) even if 18 and 58 patients had >30 and >30 Gy of single and cumulative lung doses, respectively. The estimated lung dose in patients with radiation pneumonitis ranged from 29.12 to 38.14 Gy in this study. The 30 Gy cutoff value should be reconsidered. For resin ^90^Y-microspheres, body surface area (BSA) method and partition dosimetry are commonly used in western and in eastern countries, respectively. In BSA method, LSF >20% is an absolute contraindication and dose reductions of 20% and 40% are recommended if LSF exceeds 10% or 15%, respectively (3, 4). In partition dosimetry, 25 Gy of the estimated lung dose is considered as an upper limit. Lung mass was previously regarded as 1,000 g. Lung mass was set as 800 and 600 g for men and women, respectively, for the Asian population since 2019 as recommended by the manufacturer (4). In all patients treated with resin ^90^Y-microspheres in this study, lung dose was recalculated with reduced lung mass (800 and 600 g for men and women, respectively). Three patients had radiation pneumonitis with a low estimated lung dose (13.21–21.01 Gy). These three patients did not have any underlying lung disease such as emphysema and did not undergo chemotherapy.

Six patients had estimated lung dose above the recommended lung dose limit in our series. In early study period, some patients were treated using glass ^90^Y-microsphere with predicted lung dose above 30 Gy, referring to Salem’s article (10) which had reported the safety of radioembolization with high predicted lung dose (patient no. 3, 4 and 5). In late study period, the recommended lung dose limit has been strictly followed. Two patients (patient no. 9 and 10) were treated using resin ^90^Y-microsphere before the revised recommendation by the manufacturer was applied, when the suggested lung dose limit was 30Gy and lung mass was set as 1,000g. Patient no. 13 was treated after embolization of arteriovenous shunt, as mentioned above.

Current dosimetry has limitations; planar scintigraphy using ^99m^Tc-MAA is not adequate to simulate biodistribution of ^90^Y-microsphere, especially in lung (11). Individual lung mass is not taken into account in these models. In addition, the lung dose limitations proposed by manufacturers are not validated with proper methodology.Thus, radiation pneumonitis after radioembolization seems to be able to occur, even though the lung dose limitation suggested by the manufacturers was followed in most patients. Consequently, the authors modified the lung dose cutoff value (i.e., 25 Gy for men and 20 Gy for women with glass ^90^Y-microspheres and 20 Gy with resin ^90^Y-microspheres).

This study has several limitations. First, the dosimetry between glass and resin ^90^Y-microspheres is different. Lung mass was considered as 1,000 and 600–800 g for glass and resin ^90^Y-microspheres, respectively. Second, superselective radioembolization *via* multiple target vessels is a common form of daily clinical practice. However, ^99m^Tc-MAA was injected into the lobar hepatic artery in most cases. Thus, the different injection sites of radioactive ^90^Y-microspheres and ^99m^Tc-MAA may affect the lung dose.

In conclusion, the present study suggests that radiation pneumonitis after radioembolization may occur even though the manufacturer’s instructions are followed, and the recommended cutoff value of the estimated lung dose may be adjusted to a slightly lower value.

## Data availability statement

The raw data supporting the conclusions of this article will be made available by the authors, without undue reservation.

## Ethics statement

The studies involving human participants were reviewed and approved by Institutional Review Board, Severance Hospital, Yonsei University Health System. Written informed consent for participation was not required for this study in accordance with the national legislation and the institutional requirements.

## Author contributions

Guarantor of integrity of the entire study: GK. Study concepts and design: H-CK. Literature research: H-CK, GK. Clinical studies: H-CK, GK. Data analysis: H-CK, GK. Manuscript preparation: H-CK. Manuscript editing: GK. All authors contributed to the article and approved the submitted version.
